# Frozen section analysis and sentinel lymph node biopsy in well differentiated thyroid cancer

**DOI:** 10.1186/1916-0216-42-48

**Published:** 2013-09-11

**Authors:** Yelda Jozaghi, Keith Richardson, Sumeet Anand, Alex Mlynarek, Michael P Hier, Véronique-Isabelle Forest, Eyal Sela, Michael Tamilia, Derin Caglar, Richard J Payne

**Affiliations:** 1 Department of Otolaryngology Head and Neck Surgery, McGill University Thyroid Cancer Center, 3755 Côte Ste-Catherine, Montreal, PQ, Canada; 2 Department of Endocrinology and Metabolism, Sir Mortimer B. Davis Jewish General Hospital, Montreal, PQ, Canada; 3 Department of Pathology, Sir Mortimer B. Davis Jewish General Hospital, Montreal, PQ, Canada

## Abstract

**Background:**

The aim of this study is to prospectively review the role of sentinel lymph node (SLN) biopsy in the management of well differentiated thyroid carcinoma (WDTC), and to determine the efficacy of intraoperative frozen section analysis at detecting SLN metastasis and central compartment involvement.

**Methods:**

The SLN biopsy protocol using 1% methylene blue was performed in 300 patients undergoing thyroidectomy for WDTC. A limited pretracheal central compartment neck dissection (CCND) was performed on all patients. Lymph nodes staining blue were considered as SLN’s. Both frozen and permanent section analyses were performed.

**Results:**

SLN’s with metastasis were found in 14.3% (43/300) of cases. Of this, 11% (33/300) were positive on intraoperative frozen section analysis. Frozen section results failed in predicting central compartment involvement in 15 cases (5%) whereas central neck compartment involvement was missed in 5 cases (1.7%) when based on permanent section results. On frozen section analysis, the sensitivity, specificity, positive predictive value and negative predictive value (95% CI) of our SLN biopsy technique aiming to remove all disease from the central compartment was 68.8% (53.6-80.9), 100% (98.1-100), 100% (87.0-100) and 94.4% (90.7-96.7) respectively with P < 0.0001. On permanent section analysis, the values were 89.6% (76.6-96.1), 100% (98.1-100), 100% (89.8-100), and 98.1% (95.3-99.3) with P < 0.0001.

**Conclusion:**

This data series demonstrates that patients with WDTC have positive SLN’s in 14.3% of cases. Moreover, when the SLN’s are negative for metastasis on frozen section, the central compartment was disease-free in 94.4% of cases. Finally, this study shows that 23.3% of positive SLN’s were false negatives on intraoperative frozen section. According to this data, SLN involvement is an accurate predictor of central compartment metastasis, however surgeons should use caution when relying on intraoperative frozen section to determine whether to perform a CCND.

## Introduction

Surgical management of patients with well differentiated thyroid carcinoma (WDTC) remains controversial. Though few will question the prognostic value of a therapeutic neck dissection in the context of clinically apparent nodal involvement, its role in the management of occult cervical lymph node metastasis in well differentiated thyroid carcinoma (WDTC) is the source of the debate [1,2]. Inherent risks of permanent hypoparathyroidism and vocal cord paresis have swayed against the adoption of routine prophylactic central compartment neck dissection (CCND) as standard management in the context of occult metastasis [2-5]. However, many thyroid surgeons will argue in favour of prophylactic CCND given an incidence of lymph node metastasis reported to be as high as 90% and low rates of morbidity in experienced hands [3,6-9]. Accordingly, an accurate SLNB technique, if found to be effective, could prove to be a valuable tool in the surgical management patients with WDTC.

Sentinel lymph node biopsy has become a widely adopted technique in the surgical management of melanoma and early stage breast carcinoma. SLNB techniques have also been proposed and are currently being investigated for additional tumor types including gynaecological malignancies [10], squamous cells carcinoma of the head and neck [11,12], colorectal cancer [13] and thyroid cancer [6,14]. The notion of sentinel lymph node biopsy (SLNB) relies on the principle of orderly progression of metastasis within a lymphatic basin [15]. The sentinel lymph node is defined as the first lymph node draining a regional lymphatic basin from a primary tumor. If the sentinel lymph node is found to be positive for metastasis, there may have had metastatic spread to the remainder of the lymphatic basin. An accurate SLNB technique is of particular relevance for patients who are found to be SLN negative, in which case the lymphatic basin is considered to be disease-free and the patients, in the case of WDTC, can be spared of CCND and its associated morbidities.

A SLN technique involving frozen section evaluation allows for a surgeon to assess the necessity for a CCND at the time of the initial surgery and to avoid a potentially more difficult reoperation of the central neck. The aim of the current study is to prospectively review the role of sentinel lymph node biopsy in the management of well differentiated thyroid carcinoma, and to determine the efficacy of intraoperative frozen section analysis at detecting SLN metastasis and central compartment involvement.

## Methods

### Patients

This prospective study involves 300 patients who were selected from the three adult teaching hospitals that are part of McGill University Cancer Center in Montreal, Quebec, Canada. Over a 3-year period, from June 2009 to June 2012, patients undergoing thyroid resection with results suspicious for thyroid carcinoma on fine-needle aspiration cytology (FNAC) were asked to participate in this study. Exclusion criteria included medullary and anaplastic thyroid cancer, benign thyroid disease, a history of previous thyroid surgery, pregnancy or active breastfeeding, and clinically evident local or distant metastasis. Written informed consent was obtained from all candidates as per the requirements of McGill University’s ethics review board.

### Surgical technique

**Figure 1:**
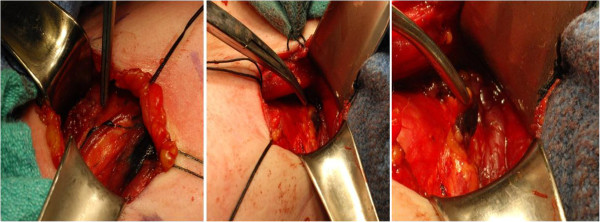
Blue staining lymphatic channels appear moments after injection of the methylene dye and lead towards the SLN within the central neck.

Following intra-operative exposure of the thyroid nodule by lateralization of the strap muscles, a 27 gauge tuberculin syringe was used to inject a total of 0.2 cc’s of 1% methylene blue dye peritumorally in all four quadrants within the thyroid parenchyma. Following the injection, 1 minute was allotted for the diffusion of the dye. Lymphatic channels staining blue (Figure 1) were traced into the central neck compartment. Lymph nodes staining blue, if present, were considered SLN’s and were harvested. Both frozen and permanent section analyses were performed. No attempt was made to identify SLN’s outside of the central neck compartment. The operative procedure was then performed as planned and followed by a limited pretracheal CCND independantly of frozen section results. The study includes all patients with confirmed thyroid carcinoma on final pathological results.

## Results

Three hundred (N = 300) patients were found to meet the inclusion criteria for the study and had confirmed thyroid carcinoma on final pathological study. The study includes 256 women and 44 men with an average age of 48.9 years (SD 13.6). The distribution of the final thyroid pathology is found in Table 1.

**Table 1:** Distribution of final thyroid gland pathology

	**Final pathology**	**N° of patients**
Conclusive fnac results	Papillary thyroid carcinoma	115
	Papillary microcarcinoma	19
	Follicular carcinoma*	4
Inconclusive fnac results	Papillary thyroid carcinoma	75
	Papillary microcarcinoma	76
	Follicular carcinoma	11

*These patients had a preoperative fine needle aspiration cytology result positive for HBME; and were pre-operatively considered to be a follicular variant of a papillary carcinoma.

Altogether, SLN’s with metastasis were found in 14.3% (43/300) of cases. Of this, 11% (33/300) were positive on intraoperative frozen section analysis. Frozen section results failed in predicting central compartment involvement in 15 cases (5%) whereas central neck compartment involvement was missed in 5 cases (1.7%) when based on permanent section results. On frozen section analysis (Table 2), the sensitivity, specificity, positive predictive value and negative predictive value (95% CI) of our SLN biopsy technique aiming to remove all disease from the central compartment was 68.8% (53.6-80.9), 100% (98.1-100), 100% (87.0-100) and 94.4% (90.7-96.7) respectively with P < 0.0001. On permanent section analysis (Table 3), the values were 89.6% (76.6-96.1), 100% (98.1-100), 100% (89.8-100), and 98.1% (95.3; 99.3) with P < 0.0001.

**Table 2:** Fisher exact contingency table- frozen section analysis

	**(+) Metastasis**	**(−) Metastasis**	**Total**	
(+)SLN	33	0	33	PPV = 100.0%
(−) SLN	15	252	267	NPV = 94.4%
Total	48	252	300	
	Sensitivity = 68.8%	Specificity = 100.0%		

*(+)SLN* sentinel lymph node positive for metastasis; *(-)SLN* sentinel lymph node negative for metastasis; *(+)Metastasis* Central compartment sample involved with metastasis;

*(-) Metastasis* Central compartment sample negative for metastasis.

**Table 3:** Fisher exact contingency table- permanent section analysis

	**(+) Metastasis**	**(−) Metastasis**	**Total**	
(+)SLN	43	0	43	PPV = 100.0%
(−) SLN	5	252	257	NPV = 98.1%
Total	48	252	300	
	Sensitivity = 89.6%	Specificity = 100.0%		

*(+)SLN* sentinel lymph node positive for metastasis; *(-)SLN* sentinel lymph node negative for metastasis; *(+)Metastasis* Central compartment sample involved with metastasis;

*(-) Metastasis* Central compartment sample negative for metastasis.

Patients were then further separated into 2 categories (Table 4): those with pre-operative FNAC that confirmed papillary thyroid carcinoma and those with inconclusive FNAC results, but final pathological findings demonstrating carcinoma of the thyroid gland. FNAC results were considered inconclusive when results indicated follicular lesions with various levels of atypia, hurthle cell lesions, and samples insufficient for diagnosis.

**Table 4:** Frozen and permanent section analyses of different patient subgroups

**Type of analysis**	**N**	**sensitivity**	**95% CI**	**specificity**	**95% CI**	**PPV**	**95% CI**	**NPV**	**95% CI**	**p-value**
FS sum	300	68.8	53.6-80.9	100	98.1-100	100	87.0-100	94.4	90.7-96.7	<0.0001
PS sum	300	89.6	76.6-96.1	100	98.1-100	100	89.8-100	98.1	95.3-99.3	<0.0001
FS, fnac (+)	138	70.7	54.3-83.4	100	95.3-100	100	85.4-100	89.0	81.2-93.9	<0.0001
PS, fnac (+)	138	90.2	75.9-96.8	100	95.3-100	100	88.3-100	96.0	89.6-98.7	<0.0001
FS, fnac (−)	162	57.1	20.2-88.2	100	97.0-100	100	39.6-100	98.1	94.1-99.5	<0.0001
PS, fnac (−)	162	85.7	42.0-99.2	100	97.0-100	100	51.7-100	99.4	95.9-100	<0.0001

*FS* frozen section, *PS* permanent section, *sum* summary analysis, *fnac* (+), conclusive pre-operative fine-needle aspiration cytology, *fnac* (−) inconclusive fine-needle aspiration cytology, *CI* confidence interval, *PPV* positive predictive value, *NPV* negative predictive value.

## Discussion

Keleman et al., in a study looking at 17 cases, were the first to report the use of SLN biopsy in the context of thyroid carcinoma [16]. However, in both this study and a similar following study by Haigh and Giuliano, a control neck dissection was not carried out and, as such, did not allow for the calculation of false negative rates, PPV’s, or NPV’s [17]. A control lateral and central neck dissection was performed in a study by Fukui et al. [18]. A SLN was found in 21 of the 22 patients, and prediction of disease status was accurate 19 of the 21 patients (90%). Finally, in a more recent study Cunningham et al. performed a retrospective review of 211 patients and concluded that SLNB is a feasible and safe technique that may allow to identify patients who can benefit from a CCND [19].

The clinical utility of a proper SLN technique with frozen section relies on its ability to predict with both high sensitivity and specificity potential metastatic involvement of a lymphatic basin. In this study, we aimed to assess not only the value of such a technique for patients undergoing surgery for cancer of the thyroid gland, but an effort was also made to evaluate the utility of this technique within subgroups of patients.

### Global study

**Figure 2:**
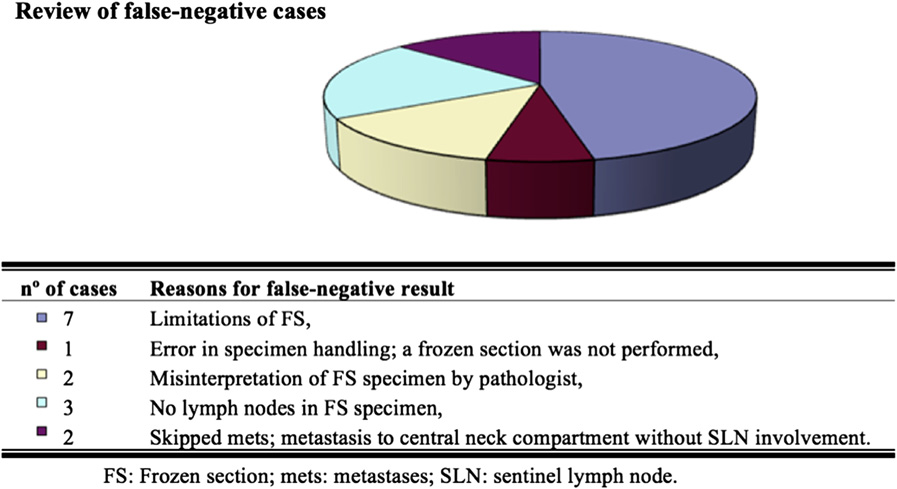
Review of false-negative cases.

First, a global analysis of all patients meeting the inclusion criteria was performed. Results revealed a significant false-negative rate of 31.3% and a 20.8% gap between sensitivity results based on frozen and permanent section analyses. Given the importance of a high sensitivity in identifying patients who can be spared of a central neck dissection, all 15 false-negative cases are herein duly reviewed in an attempt to explain the potential shortcomings of our SLN technique (Figure 2).

Based on this data series, the main contributor to the false-negative cases involves an increased difficulty in diagnosing metastasis to a lymph node on frozen section as compared to permanent section. Of the 15 false negative cases in which the SLN specimen was judged to be metastasis-free at the time of frozen section, 10 cases were revealed to harbor metastasis upon permanent section. Though 3 of these were explained by inadequate specimen handling, and frank misinterpretation of the histological section; the 7 remaining instances were reflective of the inherent limitations of a frozen section study. However, it is of note that in 9 of these 10 cases, the sentinel lymph node was the only location within the central neck that was addressed to which there had been metastasis. In other words, despite not being able to demonstrate metastatic involvement at the time of the operation, our SLN technique was in and of itself effective in rendering a disease-free neck compartment in 9 of 10 false-negative cases in the area that was addressed. Presumably, these cases could be considered as early metastases involving minute portions of the SLN, which would predispose to sampling errors at the time of frozen section.

An additional short-coming of our method is most appreciable when observing the 5 false-negative cases on permanent section analysis. In fact, merely 2 of these represented cases in which the SLN was disease-free while there had been metastatic spread to the central neck compartment. In the 3 additional cases, the specimen sent for frozen section did not contain a SLN. In fact, had we rejected these from the analysis, our SNL technique would have been associated with a sensitivity of 95.6% on permanent section analysis and 73.3% on frozen section analysis.

Altogether, a summary analysis of all patients included in the study allows us to conclude that our SLN technique, serving as a surrogate diagnostic tool for central neck compartment metastatic involvement, is 73.3% sensitive and 100% specific based on frozen section results when a SLN is found. Moreover, the apparent low sensitivity is a direct result of limitations a frozen section study. Nevertheless, the SLN technique when considered as treatment arm for patients with central neck compartment metastasis and a detectable SLN is 93.3% (42/45) efficacious in providing a disease-free neck for the area that was addressed. In this case, the non-concordance between the failure rate of our technique (6.7%) and its associated false-negative rate is accounted for by the 9 false-negative cases in which the SLN was the only site of metastasis.

### Subgroup study

That said, further subgroup analysis was performed in order to assess for confounding. On one hand, the analysis of the positive FNAC subgroup reveals sensitivity and specificity results that are slightly stronger than the global assessment (Table 4), and an associated efficacy of 92.3% (36/39) in patients with a detectable SLN. One hundred thirty eight patients were included in this category, of which 41 were found to have metastatic involvement of the central neck lymphatic basin. There were 12 false negative results on frozen section analysis, 5 instances in which the central compartment was not rendered disease-free following our SLN technique, but only 2 of these cases had detectable SLN’s in the in the SLN specimen recovered.

On the other hand, the analysis of the subgroup with inconclusive pre-operative FNAC results reveals much weaker results than those determined in the global study and the FNAC (+) subgroup. Of the total 162 patients in this category, merely 7 were found to have metastatic involvement of the central compartment, with 3 false-negatives resulting in a 57.1% sensitivity value and a very wide 95% CI (Figure 2). Thus, it would seem that the utility of a SLNB technique in patients without a conclusive pre-operative FNAC result would be highly questionable.

## Conclusion

This data series demonstrates that patients with WDTC have positive SLN’s in 14.3% of cases. This study also shows that 23.3% of positive SLN’s were false negatives on intraoperative frozen section. Nevertheless, when the SLN’s are negative for metastasis on frozen section, the central compartment was disease-free in 94.4% of cases. Accordingly, SLN involvement is an accurate predictor of central compartment metastasis; however surgeons should use caution when relying on intraoperative frozen section to determine whether to perform a CCND. Best results were associated with patients with conclusive pre-operative FNAC (demonstrating or highly suspicious for carcinoma of the thyroid gland) and those for which the SLN specimen contained a detectable lymph node. Despite a sensitivity of 70.7% in this category of patients, our SLN technique was associated with a 92.3% efficacy in rendering a disease-free central neck in patients with metastatic involvement and a detectable SLN.

For many surgeons in our thyroid cancer center, the sentinel lymph node technique has become a mainstay of treatment in patients undergoing thyroidectomy for suspected thyroid cancer. It has proven to be an effective tool in reflecting cancer status of the central neck and it is the only tool available that may help avoid unnecessary neck dissections and its associated complications in patients in whom the status of the lymphatic basin is unknown.

## Competing interests

The authors declare that they have no competing interests.

## Authors’ contributions

YJ and KR were involved in manuscript drafting, data interpretation and analysis. SA, VIF, and ES were involved in manuscript review and editing. AM, MPH, and MT were involved in patient recruitment, manuscript review and editing. DC was involved in the review of the pathological samples and in the review of pathological results in false negative cases. RJP was the study supervisor, he performed the sentinel lymph node biopsies, central neck dissections and thyroidectomies; he was involved in manuscript drafting, data interpretation, manuscript review and editing. All authors read and approved the final manuscript.

## References

[B1] Hughes CJ Shaha AR Shah JP Loree TR Impact of lymph node metastasis in differentiated carcinoma of the thyroid: A matched-pair analysis Head Neck 1996 18 2 127 132 10.1002/(SICI)1097-0347(199603/04)18:2<127::AID-HED3>3.0.CO;2-3 8647677

[B2] Anand SM Gologan O Rochon L Tamilia M How J Hier MP The role of sentinel lymph node biopsy in differentiated thyroid carcinoma Arch Otolaryngol Head Neck Surg 2009 135 12 1199 1204 10.1001/archoto.2009.190 20026816

[B3] Henry JF Gramatica L Denizot A Kvachenyuk A Puccini M Defechereux T Morbidity of prophylactic lymph node dissection in the central neck area in patients with papillary thyroid carcinoma Langenbecks Arch Surg 1998 383 2 167 169 10.1007/s004230050111 9641892

[B4] Pereira JA Jimeno J Miquel J Iglesias M Munné A Sancho JJ Nodal yield, morbidity, and recurrence after central neck dissection for papillary thyroid carcinoma Surgery 2005 138 6 1095 1101 10.1016/j.surg.2005.09.013 16360396

[B5] Stoeckli SJ Pfaltz M Steinert H Schmid S Sentinel lymph node biopsy in thyroid tumors: A pilot study Eur Arch Otorhinolaryngol 2003 260 7 364 368 10.1007/s00405-003-0594-y 12937912

[B6] Dixon E McKinnon JG Pasieka JL Feasibility of sentinel lymph node biopsy and lymphatic mapping in nodular thyroid neoplasms World J Surg 2000 24 11 1396 1401 10.1007/s002680010231 11038213

[B7] Dzodic R Sentinel lymph node biopsy may be used to support the decision to perform modified radical neck dissection in differentiated thyroid carcinoma World J Surg 2006 30 5 841 846 10.1007/s00268-005-0298-0 16680598

[B8] Shaha AR Thyroid cancer: Extent of thyroidectomy Cancer Control 2000 7 3 240 245 10832110 10.1177/107327480000700303

[B9] Hamming JF Van de Velde CJH Goslings BM Fleuren GJ Hermans J Delemarre JF Peroperative diagnosis and treatment of metastases to the regional lymph nodes in papillary carcinoma of the thyroid gland Surg Gyn Obs 1989 169 2 107 114 2667172

[B10] Makar APH Scheistroen M Van Den Weyngaert D Tropé CG Surgical management of stage I and II vulvar cancer: The role of the sentinel node biopsy Review of literature. Int J Gynecol Cancer 2001 11 4 255 262 10.1046/j.1525-1438.2001.011004255.x 11520362

[B11] Pitman KT Ferlito A Devaney KO Shaha AR Rinaldo A Sentinel lymph node biopsy in head and neck cancer Oral Oncol 2003 39 4 343 349 10.1016/S1368-8375(02)00086-6 12676253

[B12] Taylor RJ Wahl RL Sharma PK Bradford CR Terrell JE Teknos TN Sentinel node localization in oral cavity and oropharynx squamous cell cancer Arch Otolaryngol Head Neck Surg 2001 127 8 970 974 10.1001/archotol.127.8.970 11493208

[B13] Saha S Seghal R Patel M Doan K Dan A Bilchik A A multicenter trial of sentinel lymph node mapping in colorectal cancer: Prognostic implications for nodal staging and recurrence Am J Surg 2006 191 3 305 310 10.1016/j.amjsurg.2005.10.028 16490536

[B14] Pelizzo MR Boschin IM Toniato A Bernante P Piotto A Rinaldo A The sentinel node procedure with Patent Blue V dye in the surgical treatment of papillary thyroid carcinoma Acta Otolaryngol 2001 121 3 421 424 10.1080/000164801300103012 11425213

[B15] Balasubramanian SP Harrison BJ Systematic review and meta-analysis of sentinel node biopsy in thyroid cancer Br J Surg 2011 98 3 334 344 10.1002/bjs.7425 21246517

[B16] Kelemen PR Van Herle AJ Giuliano AE Sentinel lymphadenectomy in thyroid malignant neoplasms Arch Surg 1998 133 3 288 292 10.1001/archsurg.133.3.288 9517742

[B17] Haigh PI Giuliano AE Sentinel lymph node dissection for thyroid malignancy Recent Results Cancer Res 2000 157 201 205 10.1007/978-3-642-57151-0_17 10857173

[B18] Fukui Y Yamakawa T Taniki T Numoto S Miki H Monden Y Sentinel lymph node biopsy in patients with papillary thyroid carcinoma Cancer 2001 92 11 2868 2874 10.1002/1097-0142(20011201)92:11<2868::AID-CNCR10129>3.0.CO;2-I 11753960

[B19] Cunningham DK Yao KA Turner RR Singer FR Van Herle AR Giuliano AE Sentinel lymph node biopsy for papillary thyroid cancer: 12 Years of experience at a single institution Ann Surg Oncol 2010 17 11 2970 2975 10.1245/s10434-010-1141-x 20552407

